# Salicyl­aldehyde–4-(dimethyl­amino)­pyridine (1/1)

**DOI:** 10.1107/S1600536810034185

**Published:** 2010-08-28

**Authors:** Chuttree Phurat, Thapong Teerawatananond, Nongnuj Muangsin

**Affiliations:** a Research Centre of Bioorganic Chemistry, Department of Chemistry, Faculty of Science, Chulalongkorn University, Bangkok, 10330, Thailand

## Abstract

In the title compound, C_7_H_10_N_2_·C_7_H_6_O_2_, the components are linked by an O—H⋯N hydrogen bond. The mean planes of two mol­ecules form a dihedral angle of 78.68 (5)°. The crystal packing exhibits weak non-classical C—H⋯O contacts.

## Related literature

For background to hydrogen bonding in crystal engineering, see: Bosch (2010 ▶); Desiraju (1989 ▶); Lehn (1995 ▶). For related structures, see: Bosch (2010 ▶); Vembu *et al.* (2003 ▶); Lo & Ng (2009 ▶).
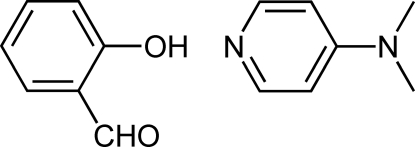


## Experimental

### 

#### Crystal data

C_7_H_10_N_2_·C_7_H_6_O_2_
*M* *_r_* = 244.29
Triclinic, 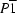

*a* = 7.540 (3) Å
*b* = 8.473 (3) Å
*c* = 10.413 (4) Å
α = 85.370 (11)°
β = 77.371 (10)°
γ = 87.203 (10)°
*V* = 646.7 (4) Å^3^
*Z* = 2
Mo *K*α radiation
μ = 0.09 mm^−1^
*T* = 296 K
0.4 × 0.4 × 0.38 mm

#### Data collection

Bruker SMART APEXII CCD area-detector diffractometer
Absorption correction: multi-scan (*SADABS*; Bruker, 2008 ▶) *T*_min_ = 0.967, *T*_max_ = 0.968
4199 measured reflections
2913 independent reflections
1882 reflections with *I* > 2σ(*I*)
*R*_int_ = 0.02

#### Refinement

*R*[*F* ^2^ > 2σ(*F* ^2^)] = 0.049
*wR*(*F* ^2^) = 0.163
*S* = 1.01
2913 reflections
166 parameters
H-atom parameters constrained
Δρ_max_ = 0.20 e Å^−3^
Δρ_min_ = −0.15 e Å^−3^

### 

Data collection: *APEX2* (Bruker, 2008 ▶); cell refinement: *SAINT* (Bruker, 2008 ▶); data reduction: *SAINT*; program(s) used to solve structure: *SHELXS97* (Sheldrick, 2008 ▶); program(s) used to refine structure: *SHELXL97* (Sheldrick, 2008 ▶); molecular graphics: *ORTEP-3* (Farrugia, 1997 ▶); software used to prepare material for publication: *SHELXL97*.

## Supplementary Material

Crystal structure: contains datablocks global, I. DOI: 10.1107/S1600536810034185/cv2756sup1.cif

Structure factors: contains datablocks I. DOI: 10.1107/S1600536810034185/cv2756Isup2.hkl

Additional supplementary materials:  crystallographic information; 3D view; checkCIF report

## Figures and Tables

**Table 1:** Hydrogen-bond geometry (Å, °)

*D*—H⋯*A*	*D*—H	H⋯*A*	*D*⋯*A*	*D*—H⋯*A*
O2—H2*A*⋯N1	0.82	1.82	2.637 (2)	174
C9—H9⋯O1^i^	0.93	2.69	3.456 (3)	140
C5—H5⋯O1^ii^	0.93	2.7	3.583 (3)	158

Symmetry codes: (i) 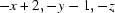; (ii) 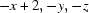.

**Fig. 1.**
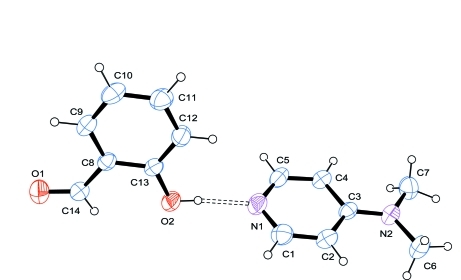
The content of asymmetric unit of the title compound showing the atom-numbering scheme. Displacement ellipsoids are drawn at the 50% probability level. Hydrogen bond is shown as a dashed line.

## supplementary crystallographic information

### Comment

Hydrogen bonding is the most important and the essential tool for both crystal engineering and supramolecular chemistry (Bosch, 2010; Desiraju, 1989 & Lehn, 1995). The non-classical C—H···N hydrogen bonds in pyridine and pyrimidine derivatives have remarkable potentials and patterns (Bosch, 2010; Desiraju, 1989; Lehn, 1995; Lo & Ng, 2009 & Vembu *et al.*, 2003;). In order to investigate the hydrogen bonding patterns of 4-(dimethylamino)pyridine, the co-crystals with various derivatives of benzaldehyde were prepared.

We report here the structure of the title co-crystal compound (Fig.1), formed from salicylaldehyde and 4-(dimethylamino)pyridine. The asymmetric unit contains one molecule of salicylaldehyde and one molecule of 4-(dimethylamino)pyridine linked by O—H···N hydrogen bond (Table 1). The mean planes of two molecules form a dihedral angle of 78.68 (5)°. The crystal packing exhibits weak non-classical C—H···O contacts (Table 1).

### Experimental

The title cocrystal was crystallized by slow evaporation from the refluxed mixture of an equimolar solution of salicylaldehyde and 4-(dimethylamino)pyridine in a solution of methanol.

### Refinement

All H-atoms were geometrically positioned and refined using a riding model, with C—H = 0.93 Å (aromatic), 0.98 Å (CH_3_) and O–H = 0.82 Å, and *U*_iso_(H) = 1.2*U*_eq_ (C) for aromatic and 1.5*U*_eq_ for O and C_methyl_.

### Crystal data

**Table:**

C_7_H_10_N_2_·C_7_H_6_O_2_	*Z* = 2
*M**_r_* = 244.29	*F*(000) = 260
Triclinic, *P*1	*D*_x_ = 1.255 Mg m^−^^3^
Hall symbol: -P 1	Mo *K*α radiation, λ = 0.71073 Å
*a* = 7.540 (3) Å	Cell parameters from 1563 reflections
*b* = 8.473 (3) Å	θ = 2.8–27.8°
*c* = 10.413 (4) Å	µ = 0.09 mm^−^^1^
α = 85.370 (11)°	*T* = 296 K
β = 77.371 (10)°	Prism, yellow
γ = 87.203 (10)°	0.4 × 0.4 × 0.38 mm
*V* = 646.7 (4) Å^3^	

### Data collection

**Table:**

Bruker SMART APEXII CCD area-detector diffractometer	2913 independent reflections
Radiation source: Mo Kα	1882 reflections with *I* > 2σ(*I*)
graphite	*R*_int_ = 0.02
φ and ω scans	θ_max_ = 28.3°, θ_min_ = 2.8°
Absorption correction: multi-scan (*SADABS*; Bruker, 2008)	*h* = −10→9
*T*_min_ = 0.967, *T*_max_ = 0.968	*k* = −5→11
4199 measured reflections	*l* = −13→13

### Refinement

**Table:**

Refinement on *F*^2^	0 restraints
Least-squares matrix: full	H-atom parameters constrained
*R*[*F*^2^ > 2σ(*F*^2^)] = 0.049	*w* = 1/[σ^2^(*F*_o_^2^) + (0.0815*P*)^2^ + 0.0644*P*] where *P* = (*F*_o_^2^ + 2*F*_c_^2^)/3
*wR*(*F*^2^) = 0.163	(Δ/σ)_max_ < 0.001
*S* = 1.01	Δρ_max_ = 0.20 e Å^−^^3^
2913 reflections	Δρ_min_ = −0.15 e Å^−^^3^
166 parameters	

### Special details

**Table:**

Geometry. All e.s.d.'s (except the e.s.d. in the dihedral angle between two l.s. planes) are estimated using the full covariance matrix. The cell e.s.d.'s are taken into account individually in the estimation of e.s.d.'s in distances, angles and torsion angles; correlations between e.s.d.'s in cell parameters are only used when they are defined by crystal symmetry. An approximate (isotropic) treatment of cell e.s.d.'s is used for estimating e.s.d.'s involving l.s. planes.

### Fractional atomic coordinates and isotropic or equivalent isotropic displacement parameters (Å^2^)

**Table:**

	*x*	*y*	*z*	*U*_iso_*/*U*_eq_	
C1	0.8429 (3)	0.2372 (2)	0.5097 (2)	0.0720 (5)	
H1	0.868	0.1401	0.5513	0.086*	
C2	0.7810 (3)	0.3587 (2)	0.58701 (17)	0.0638 (5)	
H2	0.7635	0.3422	0.6782	0.077*	
C3	0.7433 (2)	0.5084 (2)	0.52993 (15)	0.0521 (4)	
C4	0.7680 (2)	0.5182 (2)	0.39168 (16)	0.0604 (4)	
H4	0.7426	0.6132	0.3469	0.072*	
C5	0.8288 (3)	0.3893 (3)	0.32350 (18)	0.0693 (5)	
H5	0.8428	0.4003	0.2323	0.083*	
C6	0.6562 (3)	0.6217 (3)	0.74452 (19)	0.0879 (7)	
H6A	0.5548	0.5552	0.7795	0.132*	
H6B	0.6298	0.725	0.777	0.132*	
H6C	0.7624	0.5762	0.7717	0.132*	
C7	0.6590 (3)	0.7888 (3)	0.5390 (2)	0.0798 (6)	
H7A	0.7616	0.8131	0.4686	0.12*	
H7B	0.6445	0.8672	0.6024	0.12*	
H7C	0.5513	0.7885	0.5041	0.12*	
C8	0.9510 (2)	−0.18638 (19)	0.10290 (14)	0.0500 (4)	
C9	0.8248 (3)	−0.2663 (2)	0.05460 (16)	0.0603 (5)	
H9	0.8641	−0.3511	0.0029	0.072*	
C10	0.6437 (3)	−0.2223 (3)	0.08184 (19)	0.0724 (5)	
H10	0.5604	−0.2764	0.0493	0.087*	
C11	0.5875 (3)	−0.0964 (3)	0.1584 (2)	0.0754 (6)	
H11	0.4652	−0.065	0.1763	0.09*	
C12	0.7082 (2)	−0.0158 (2)	0.20895 (18)	0.0654 (5)	
H12	0.6669	0.0687	0.2605	0.078*	
C13	0.8914 (2)	−0.06070 (19)	0.18288 (14)	0.0515 (4)	
C14	1.1435 (3)	−0.2309 (2)	0.06846 (17)	0.0631 (5)	
H14	1.2235	−0.1669	0.0951	0.076*	
N1	0.8703 (2)	0.2475 (2)	0.37777 (17)	0.0724 (5)	
N2	0.6887 (2)	0.63491 (19)	0.60206 (13)	0.0636 (4)	
O1	1.2084 (2)	−0.34318 (19)	0.00855 (16)	0.0891 (5)	
O2	1.01269 (17)	0.01257 (16)	0.23295 (13)	0.0670 (4)	
H2A	0.961	0.0844	0.2765	0.101*	

### Atomic displacement parameters (Å^2^)

**Table:**

	*U*^11^	*U*^22^	*U*^33^	*U*^12^	*U*^13^	*U*^23^
C1	0.0818 (13)	0.0592 (12)	0.0816 (13)	−0.0101 (9)	−0.0299 (10)	−0.0061 (9)
C2	0.0763 (12)	0.0654 (12)	0.0532 (9)	−0.0126 (9)	−0.0203 (8)	−0.0007 (8)
C3	0.0488 (8)	0.0622 (11)	0.0477 (8)	−0.0119 (7)	−0.0121 (7)	−0.0073 (7)
C4	0.0678 (10)	0.0654 (11)	0.0503 (9)	−0.0068 (8)	−0.0163 (8)	−0.0055 (8)
C5	0.0764 (12)	0.0813 (15)	0.0542 (10)	−0.0080 (10)	−0.0162 (9)	−0.0188 (9)
C6	0.1038 (16)	0.1054 (19)	0.0546 (11)	0.0003 (13)	−0.0119 (10)	−0.0232 (11)
C7	0.0877 (14)	0.0677 (14)	0.0813 (13)	0.0053 (10)	−0.0114 (11)	−0.0126 (10)
C8	0.0633 (10)	0.0484 (9)	0.0393 (7)	−0.0072 (7)	−0.0137 (7)	0.0023 (6)
C9	0.0776 (12)	0.0578 (11)	0.0483 (9)	−0.0128 (8)	−0.0154 (8)	−0.0085 (7)
C10	0.0688 (12)	0.0883 (15)	0.0667 (11)	−0.0241 (10)	−0.0209 (9)	−0.0136 (10)
C11	0.0588 (11)	0.0952 (16)	0.0755 (12)	−0.0095 (10)	−0.0163 (9)	−0.0173 (11)
C12	0.0636 (11)	0.0681 (12)	0.0668 (11)	−0.0022 (9)	−0.0141 (8)	−0.0187 (9)
C13	0.0615 (10)	0.0502 (9)	0.0455 (8)	−0.0089 (7)	−0.0170 (7)	−0.0008 (7)
C14	0.0698 (11)	0.0637 (12)	0.0600 (10)	−0.0001 (9)	−0.0218 (8)	−0.0105 (8)
N1	0.0745 (10)	0.0704 (12)	0.0794 (11)	−0.0065 (8)	−0.0232 (8)	−0.0264 (8)
N2	0.0734 (9)	0.0667 (10)	0.0507 (8)	−0.0049 (7)	−0.0103 (7)	−0.0114 (7)
O1	0.0889 (10)	0.0822 (11)	0.1002 (11)	0.0161 (8)	−0.0239 (8)	−0.0325 (8)
O2	0.0665 (8)	0.0665 (9)	0.0756 (8)	−0.0047 (6)	−0.0244 (6)	−0.0235 (6)

### Geometric parameters (Å, °)

**Table:**

C1—N1	1.340 (3)	C7—H7B	0.96
C1—C2	1.359 (3)	C7—H7C	0.96
C1—H1	0.93	C8—C9	1.394 (2)
C2—C3	1.400 (3)	C8—C13	1.401 (2)
C2—H2	0.93	C8—C14	1.455 (3)
C3—N2	1.355 (2)	C9—C10	1.372 (3)
C3—C4	1.407 (2)	C9—H9	0.93
C4—C5	1.357 (3)	C10—C11	1.378 (3)
C4—H4	0.93	C10—H10	0.93
C5—N1	1.339 (3)	C11—C12	1.378 (3)
C5—H5	0.93	C11—H11	0.93
C6—N2	1.446 (2)	C12—C13	1.389 (3)
C6—H6A	0.96	C12—H12	0.93
C6—H6B	0.96	C13—O2	1.3452 (18)
C6—H6C	0.96	C14—O1	1.205 (2)
C7—N2	1.442 (3)	C14—H14	0.93
C7—H7A	0.96	O2—H2A	0.82
			
N1—C1—C2	124.69 (19)	C9—C8—C13	119.44 (16)
N1—C1—H1	117.7	C9—C8—C14	120.36 (16)
C2—C1—H1	117.7	C13—C8—C14	120.19 (14)
C1—C2—C3	120.29 (17)	C10—C9—C8	121.25 (17)
C1—C2—H2	119.9	C10—C9—H9	119.4
C3—C2—H2	119.9	C8—C9—H9	119.4
N2—C3—C2	122.62 (15)	C9—C10—C11	118.68 (17)
N2—C3—C4	122.36 (16)	C9—C10—H10	120.7
C2—C3—C4	115.02 (16)	C11—C10—H10	120.7
C5—C4—C3	120.10 (18)	C10—C11—C12	121.63 (19)
C5—C4—H4	120	C10—C11—H11	119.2
C3—C4—H4	120	C12—C11—H11	119.2
N1—C5—C4	124.86 (17)	C11—C12—C13	119.98 (18)
N1—C5—H5	117.6	C11—C12—H12	120
C4—C5—H5	117.6	C13—C12—H12	120
N2—C6—H6A	109.5	O2—C13—C12	121.78 (16)
N2—C6—H6B	109.5	O2—C13—C8	119.24 (15)
H6A—C6—H6B	109.5	C12—C13—C8	118.99 (15)
N2—C6—H6C	109.5	O1—C14—C8	125.87 (17)
H6A—C6—H6C	109.5	O1—C14—H14	117.1
H6B—C6—H6C	109.5	C8—C14—H14	117.1
N2—C7—H7A	109.5	C5—N1—C1	114.99 (16)
N2—C7—H7B	109.5	C3—N2—C7	120.92 (15)
H7A—C7—H7B	109.5	C3—N2—C6	121.81 (17)
N2—C7—H7C	109.5	C7—N2—C6	117.26 (16)
H7A—C7—H7C	109.5	C13—O2—H2A	109.5
H7B—C7—H7C	109.5		
			
N1—C1—C2—C3	−1.0 (3)	C9—C8—C13—O2	177.74 (14)
C1—C2—C3—N2	−177.17 (16)	C14—C8—C13—O2	−3.5 (2)
C1—C2—C3—C4	2.3 (2)	C9—C8—C13—C12	−1.9 (2)
N2—C3—C4—C5	177.77 (16)	C14—C8—C13—C12	176.90 (16)
C2—C3—C4—C5	−1.7 (2)	C9—C8—C14—O1	−6.8 (3)
C3—C4—C5—N1	−0.3 (3)	C13—C8—C14—O1	174.44 (18)
C13—C8—C9—C10	1.3 (2)	C4—C5—N1—C1	1.7 (3)
C14—C8—C9—C10	−177.51 (16)	C2—C1—N1—C5	−1.1 (3)
C8—C9—C10—C11	0.1 (3)	C2—C3—N2—C7	177.21 (17)
C9—C10—C11—C12	−0.8 (3)	C4—C3—N2—C7	−2.2 (3)
C10—C11—C12—C13	0.2 (3)	C2—C3—N2—C6	−3.3 (3)
C11—C12—C13—O2	−178.43 (16)	C4—C3—N2—C6	177.28 (17)
C11—C12—C13—C8	1.2 (3)		

### Hydrogen-bond geometry (Å, °)

**Table:**

*D*—H···*A*	*D*—H	H···*A*	*D*···*A*	*D*—H···*A*
O2—H2A···N1	0.82	1.82	2.637 (2)	174
C9—H9···O1^i^	0.93	2.69	3.456 (3)	140
C5—H5···O1^ii^	0.93	2.7	3.583 (3)	158

Symmetry codes: (i) −*x*+2, −*y*−1, −*z*; (ii) −*x*+2, −*y*, −*z*.
